# Prognostic values of a novel multi-mRNA signature for predicting relapse of cholangiocarcinoma

**DOI:** 10.7150/ijbs.38846

**Published:** 2020-01-16

**Authors:** Han Guo, Jie Cai, Xuan Wang, Bingrui Wang, Fang Wang, Xiang Li, Xiaoye Qu, Xianming Kong, Yueqiu Gao, Hailong Wu, Xuehua Sun, Qiang Xia, Xiaoni Kong

**Affiliations:** 1 Department of Liver Surgery, Renji Hospital, School of Medicine, Shanghai Jiao Tong University, Shanghai, China.; 2 Institute of Clinical Immunology, Department of Liver Diseases, Central Laboratory, ShuGuang Hospital Affiliated to Shanghai University of Chinese Traditional Medicine, Shanghai, China; 3 Shanghai Key Laboratory for Molecular Imaging, Collaborative Research Center, Shanghai University of Medicine and Health Sciences, Shanghai, China.; 4 Central Laboratory, Renji Hospital, School of Medicine, Shanghai Jiao Tong University, Shanghai, China.

**Keywords:** cholangiocarcinoma, Gene Expression Omnibus database, least absolute shrinkage and selection operator model, mRNA signature, recurrence-free survival.

## Abstract

Cholangiocarcinoma (CCA) is an epithelial cancer and has high death and recurrence rates, current methods cannot satisfy the need for predicting cancer relapse effectively. So, we aimed at conducting a multi-mRNA signature to improve the relapse prediction of CCA. We analyzed mRNA expression profiling in large CCA cohorts from the Gene Expression Omnibus (GEO) database (GSE76297, GSE32879, GSE26566, GSE31370, and GSE45001) and The Cancer Genome Atlas (TCGA) database. The Least absolute shrinkage and selection operator (LASSO) regression model was used to establish a 7-mRNA-based signature that was significantly related to the recurrence-free survival (RFS) in two test series. Based on the 7-mRNA signature, the cohort TCGA patients could be divided into high-risk or low-risk subgroups with significantly different RFS [p < 0.001, hazard ratio (HR): 48.886, 95% confidence interval (CI): 6.226-3.837E+02]. Simultaneously, the prognostic value of the 7-mRNA signature was confirmed in clinical samples of Ren Ji hospital (p < 0.001, HR: 4.558, 95% CI: 1.829-11.357). Further analysis including multivariable and sub-group analyses revealed that the 7-mRNA signature was an independent prognostic value for recurrence of patients with CCA. In conclusion, our results might provide an efficient tool for relapse prediction and were beneficial to individualized management for CCA patients.

## Introduction

Cholangiocarcinoma (CCA) is the second most common primary liver cancer worldwide 1-3. During the past few decades, incidence and high recurrence rates for all CCA were closely correlated to poor outcomes 4-6. Unfortunately, there is still no better way to accurately predict recurrence. Although TNM staging systems according to different versions of the American Joint Committee on Cancer (AJCC) had shown valuable but still insufficient for predicting relapse in different subtypes of CCA 5.

An increasing amount of evidence has demonstrated that messenger RNA (mRNA) as molecular biomarkers could promote the prognostic evaluation and identification of potential high-risk CCA patients 7, 8. For example, KRAS mutations were associated with deregulation of epidermal growth factor receptor (EGFR) and ERBB2 signaling network, derangement of genes participating in proteasomal activity could lead to poor prognosis 9. However, many genes and signal pathways were also present in hepatocellular carcinoma, and single gene as a prognostic indicator for CCA was not rigorous enough. So, our study aimed at finding a multi-mRNA model to help better predicting the relapse of CCA patients.

GEO 10 and The Cancer Genome Atlas 11 are two main public databases that provided numerous array-based and sequence-based data for researchers. By using bioinformatic methods 12, we could obtain large amounts of data quickly and conveniently. Therefore, we identified significant genes that expressed differentially between CCA samples and normal bile duct tissue or para cancerous samples in five datasets from GEO and TCGA database, respectively. Then, we utilized the least absolute shrinkage and selection operator (LASSO) regression model 13, 14 and built a 7-mRNA-based signature for predicting relapse. Cox regression and the time-dependent ROC curve demonstrated that this 7-mRNA-based signature had an excellent prediction for RFS. In addition, gene ontology (GO) enrichment analysis and Kyoto Encyclopedia of Genes and Genomes (KEGG) pathway analysis were performed for discovering essential marker and pathways. All of these may provide an efficient method to judge recurrence rate and was beneficial to individualized management for CCA patients.

## Materials and methods

### Preparation of CCA datasets

The gene expression data of CCAs were downloaded from Gene Expression Omnibus (GEO) and The Cancer Genome Atlas (TCGA) database. There were five appropriate CCA datasets from the GEO database (GSE76297, GSE32879, GSE26566, GSE31370, and GSE45001) met the following criteria: a). a total of more than 10 samples, including both tumor and non-tumor samples; b) annotated genes accounting for more than 90% of the total transcriptomes (n > 17000); and c) the number of differentially expressed genes (DEGs) more than 100. Details of these five datasets were listed in Table 1. In addition, the TCGA database provided gene expression profiles from RNA-seq and corresponding clinical information in 36 CCA patients with RFS status. The online analytical tool GEO2R 12 was used to screen out DEGs between CCA and non-tumor samples in the GEO database, and we obtained DEGs from the TCGA database by using R package “edgeR”. Here, genes with adjusted p-value < 0.01 and fold change (FC) >1.5 or <-1.5 were considered as DEGs.

Three gene expression profiles were utilized to recognize DEGs between tumor tissues and normal intrahepatic bile duct tissues or non-tumor tissues (T/N), and the other three gene expression profiles were used to recognize DEGs between tumor tissues and para-cancerous tissues (T/P). Next, overlapping analysis of these DEGs was conducted by website imageGP, DEGs within 2 series or more were regarded as credible DEGs in each Venn diagram. Finally, 194 DEGs among TvsN, TvsP and TCGA were identified, which including 87 up-regulated genes and 107 down-regulated genes.

### GO enrichment analysis and KEGG pathway analysis

GFO analysis is a common genes and gene products annotating method, including biological processes (BP), cellular component (CC), molecular function (MF). The Kyoto Encyclopedia of Genes and Genomes (KEGG) database is a knowledge base for systematic analysis, annotation, and visualization of gene functions. In our study, the R package “clusterprofiler” was used to provide functional classification, and KEGG pathway, of the 87 up-regulated and 107 down-regulated DEGs, respectively. We listed the top 10 of all terms in every category, p < 0.05 was set as the cutoff point.

### Establishment of the LASSO regression model

For these 194 candidate mRNAs, the optimal cutoff value of each mRNA was generated based on receiver operating characteristic (ROC) curve, and the area under the curve (AUC), sensitivities and specificities of these mRNAs were also obtained. Next, there were 127 genes with AUC ≥ 0.55 remained. According to the cutoff value, 36 patients of the TCGA database were classified into high- or low-expression status according to each mRNA. Based on the expression status data of these 127 DEGs, we constructed LASSO COX regression models with the R package “glmnet”. The least absolute shrinkage and selection operator (LASSO) is a most famous method for analyzing survival data, and especially suitable for analyzing gene expression profile, which has higher dimensionality, smaller sample size and strongly relevant variables^15^, ^16^. The “glmnet” package returned a sequence of models for us, the value of the tuning parameter λ was negatively associated with the complexity of the model and the value of deviance. When the value of the invisible λ increased from left to right, the number of nonzero coefficients increased accordingly. Ten-time cross validations were used to determine the optimal values of λ and a value λ = 0.20770 with log (λ) = -0.68256 was chosen by 10-fold cross-validation via minimum criteria; a vertical line was drawn at L1 norm=2.388, which corresponds to the optimal value = 0.20770. However, the results of the λ value might be slightly variable during different times of analysis. So, 10-fold cross-validation was running up to 100 times and the cross-validated errors were averaged.

### Validation in Clinical CCA specimens

Between January 1, 2012, and December 30, 2017, the human CCA tissues were obtained from the Department of Liver Surgery, Ren Ji Hospital, Shanghai Jiaotong University. Protocol and free of written informed consent were approved by the ethical review committee of Renji Hospital, School of Medicine, Shanghai Jiaotong University.

We excluded patients for the following criteria: combination with other tumors, perioperative mortality, preoperative radiotherapy and chemotherapy, conservative treatment and incomplete data. Finally, we obtained 44 patients' tissues and all tissues were pathologically confirmed. The clinicopathological features of the Ren Ji cohort were listed in Table S1. Tumor staging was assessed according to the 8th edition staging classification system of AJCC 17.

Follow up information of these CCA patients has received check-ups every 2-3 months during the first 2 years and every 3-6 months until May 2018. The RFS was calculated from the date of tumor resection until the detection of tumor recurrence, death from a cause other than CCA, or the last follow-up visit.

### Quantitative real-time PCR (qRT-PCR)

Total RNA was extracted and reversed using the RNeasy Mini Kit (Qiagen, Valencia, CA) and the Revert Aid First Strand cDNA Synthesis Kit (Thermo Scientific, Rockford, IL), respectively. The expression of CD36, GGCX, UBASH3B, DBN1, PTTG1, CCNA2, SPATS2, and 18S mRNA were determined by qRT-PCR using SYBR Green PCR Master Mix, and Ct value was enrolled for data analysis. Related primers sequences were listed in Table S2. All these experiments were conducted according to the manufacture instructions.

### Statistical analysis

The statistical analysis was carried out using SPSS 17.0 and GraphPad Prism 6 software. The optimal cutoff of risk score was determined when the sensitivity and specificity in the ROC curve 18, 19 achieved optimum for predicting recurrence-free survival. With this risk score cutoff, the patients were divided into high- or low-risk groups. Recurrence-free survival analysis between high- and low-risk groups was assessed by the Kaplan-Meier analysis and compared using the log-rank test. Time-dependent ROC curves were employed to demonstrate the predictive accuracy of different variables. Univariable and multivariable Cox analyses were performed to investigate whether the gene signature was independent of other clinicopathological characteristics, and Pearson chi-squared test or Fisher's exact test was used to examine the association between the clinicopathological characteristics and 7-mRNA signature. A difference was defined as significant at P < 0.05.

## Results

### Identification of differentially expressed genes in cholangiocarcinoma from public datasets

**Figure 1:**
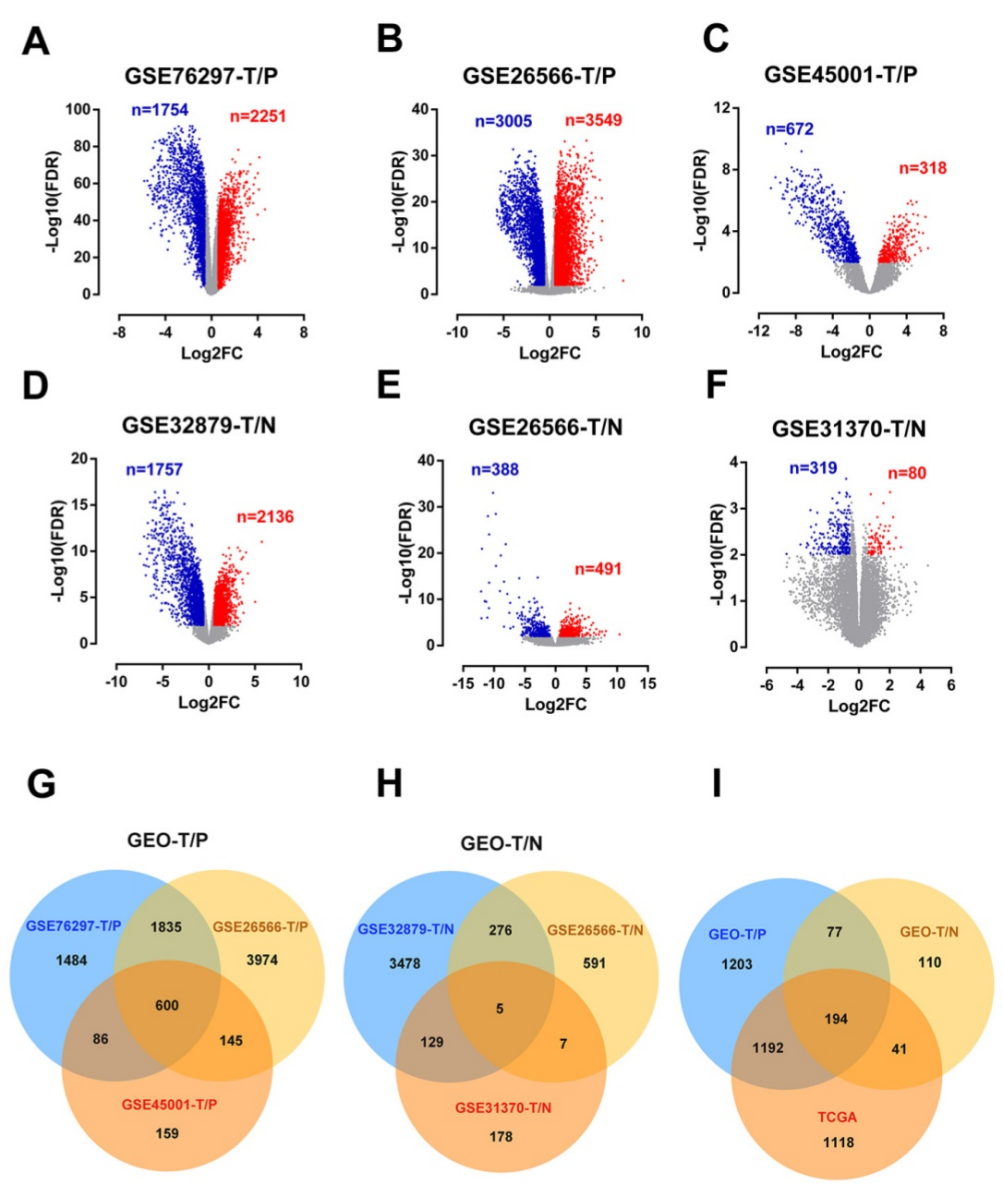
Identification of differentially expressed genes in cholangiocarcinoma from public CCA datasets. (A-F) Volcano plots of DEGs in the 5 indicated datasets. (X-axis: log2(FC); Y-axis: -log10(FDR) for each gene. Genes with FDR < 0.01 and FC >1.5 or <-1.5 were considered as DEGs in each series. Blue: down-regulated genes; Grey: non-differential genes; Red: up-regulated genes). (G-H) Overlapping analyses of DEGs in TvsP (G) and TvsN (H) groups, DEGs shared within 2 datasets or more were regarded as credible DEGs in each Venn diagram. (I) Overlapping analysis of GEO and TCGA datasets.

Detailed information of the five eligible CCA datasets meets our criteria in the GEO database (GSE76297, GSE32879, GSE26566, GSE31370, and GSE45001) were shown in Table 1. After analyzing these CCA datasets using GEO2R, 4005, 6554, 990, 3893, 879 and 399 DEGs were respectively recognized in GSE76297-T/P, GSE26566-T/P, GSE45001-T/P, GSE32879-T/N, GSE26566-T/N, GSE31370-T/N (Figure 1A-F). DEGs shared within 2 series or more were regarded as credible DEGs in each Venn diagram, and 2666 and 422 credible DEGs were recognized in T/P and T/N groups respectively (Figure 1G-H). Similarly, 2545 DEGs meet the criteria with p-value < 0.01 and FC >1.5 or <-1.5 were gathered in TCGA, including 1132 up-regulated and 1413 down-regulated genes (Figure S1). The Overlapping analysis was further performed between GEO and TCGA database, and 194 DEGs were identified, which were believed to be commonly dysregulated in CCA (Figure 1I).

**Figure 2:**
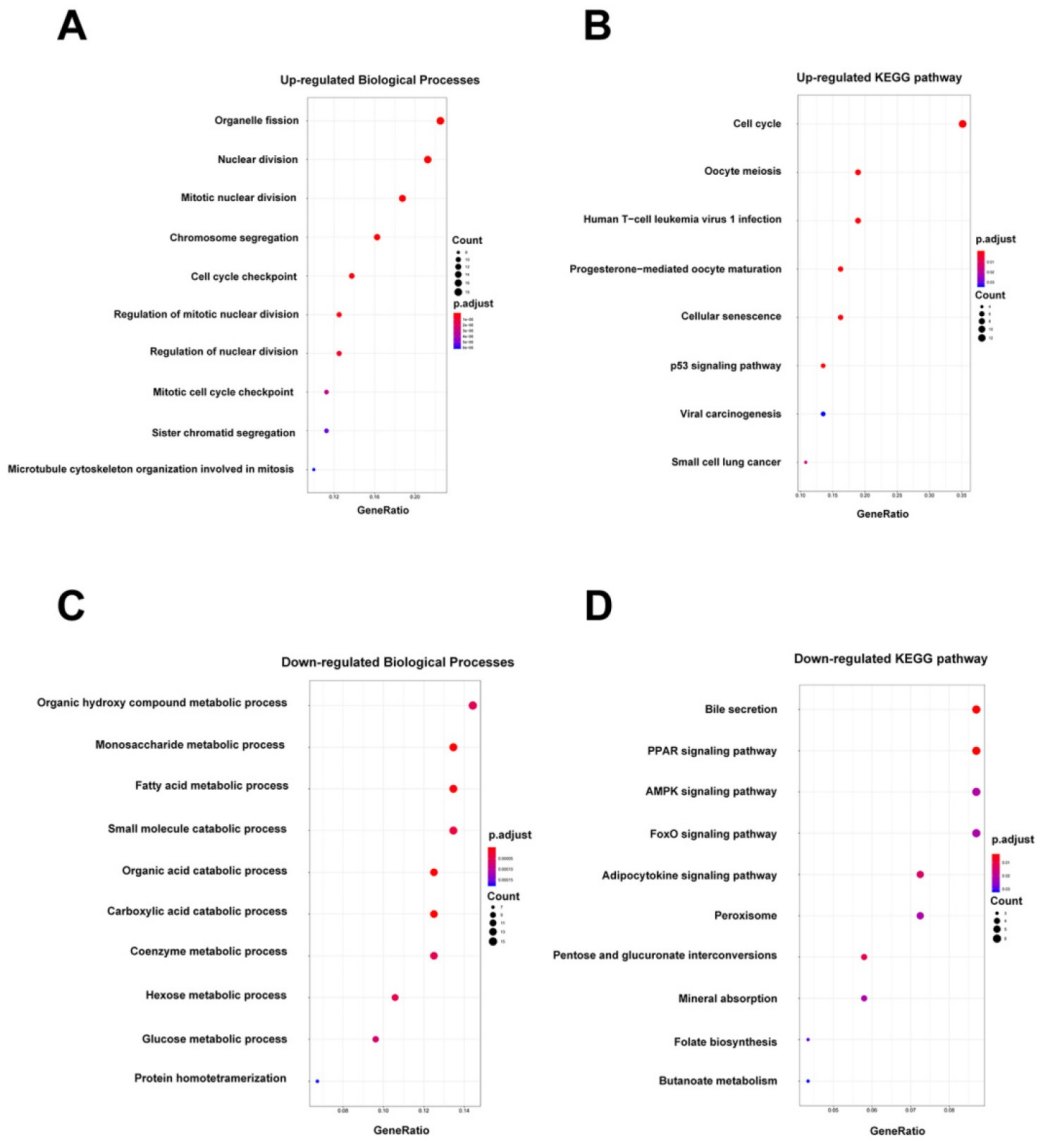
Biological processes (BP) enrichment analysis and KEGG pathway analysis. (A-B) GO biological processes (BP) enrichment analysis and KEGG pathway analysis of upregulated DEGs. (C-D) GO biological processes (BP) enrichment analysis and KEGG pathway analysis of downregulated DEGs.

In addition, GO and KEGG pathway enrichment analyses were conducted for these overlapping up- or down-regulated genes. As shown in Figure 2A-B, up-regulated genes were most enriched in organelle fission and cell cycle pathways by GO-BP and KEGG analyses, respectively. Contrarily, the organic hydroxy compound metabolic process and bile secretion pathway were respectively greatly enriched in GO-BP and KEGG analyses by down-regulated genes (Figure 2C-D). Meanwhile, enrichment analyses for cellular component (CC) and molecular functions (MF) were also performed (Figure S2).

### Construction of a 7-mRNA signature from the TCGA cohort

For these 194 candidate DEGs, the optimal cutoff point was determined when the sensitivity and specificity of the ROC curve achieved optimum. According to each mRNA cutoff value, 36 patients were classified into high or low expression status. Besides, AUC ≥ 0.55 was a restrictive condition for filtering some mRNAs that hardly had a prognostic value. Ultimately, 127 mRNAs with AUC≥0.55 were utilized to construct the LASSO COX regression model.

**Figure 3:**
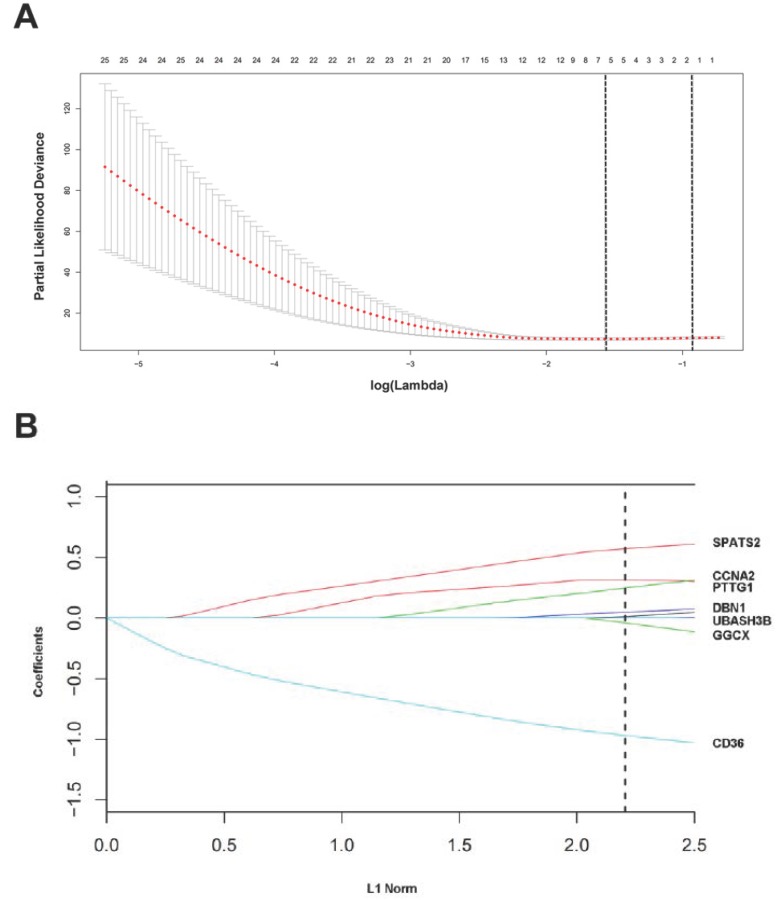
Construction of a 7-mRNA signature from the TCGA cohort. (A) 10-fold cross-validation for tuning parameter selection in the LASSO model. The dotted vertical lines are drawn at the optimal values by minimum criteria (lambda.min, left vertical dotted line) and 1-SE criteria (lambda.1se, right vertical dotted line). (B) LASSO model at optimal lambda value, 7 mRNAs with nonzero coefficients were selected.

The “glmnet” package 13, 20 returned a sequence of models for us (Figure S3A), and 10-fold cross-validations were performed to select the best one. As shown in Figure 3A, a value λ = 0.20770 with log (λ) = -0.68256 was chosen by 10-fold cross-validation via minimum criteria. However, the results of the λ value might be slightly variable during different times of analysis. So, 10-fold cross-validation was running up to 100 times and the cross-validated errors were averaged. Finally, the λ with minimum mean cross validation error was still returned about 0.20770. At this λ value, 7 mRNAs including CD36, GGCX, UBASH3B, DBN1, PTTG1, CCNA2 and SPATS2 with nonzero coefficients were selected (Figure 3B). Among them, CD36 and GGCX were down-regulated in CCA, and the other 5 genes were up-regulated. Meanwhile, person's correlation tests showed that the expression of these 7 genes was independent of each other (Figure S3B). Based on the expression status of these 7 mRNAs, a risk-score formula for RFS was constructed as follows: Risk score= (-0.96873 × expression status of CD36) + (-0.03944 × expression status of GGCX) + (0.01064 × expression status of UBASH3B) + (0.04955 × expression status of DBN1) + (0.24927 × expression status of PTTG1) + (0.31598 × expression status of CCNA2) + (0.57201 × expression status of SPATS2). In the formula, low expression status was equivalent to 0, and high expression status was equivalent to 1.

### Evaluation of the risk score formula for relapse in TCGA Cohort

**Figure 4:**
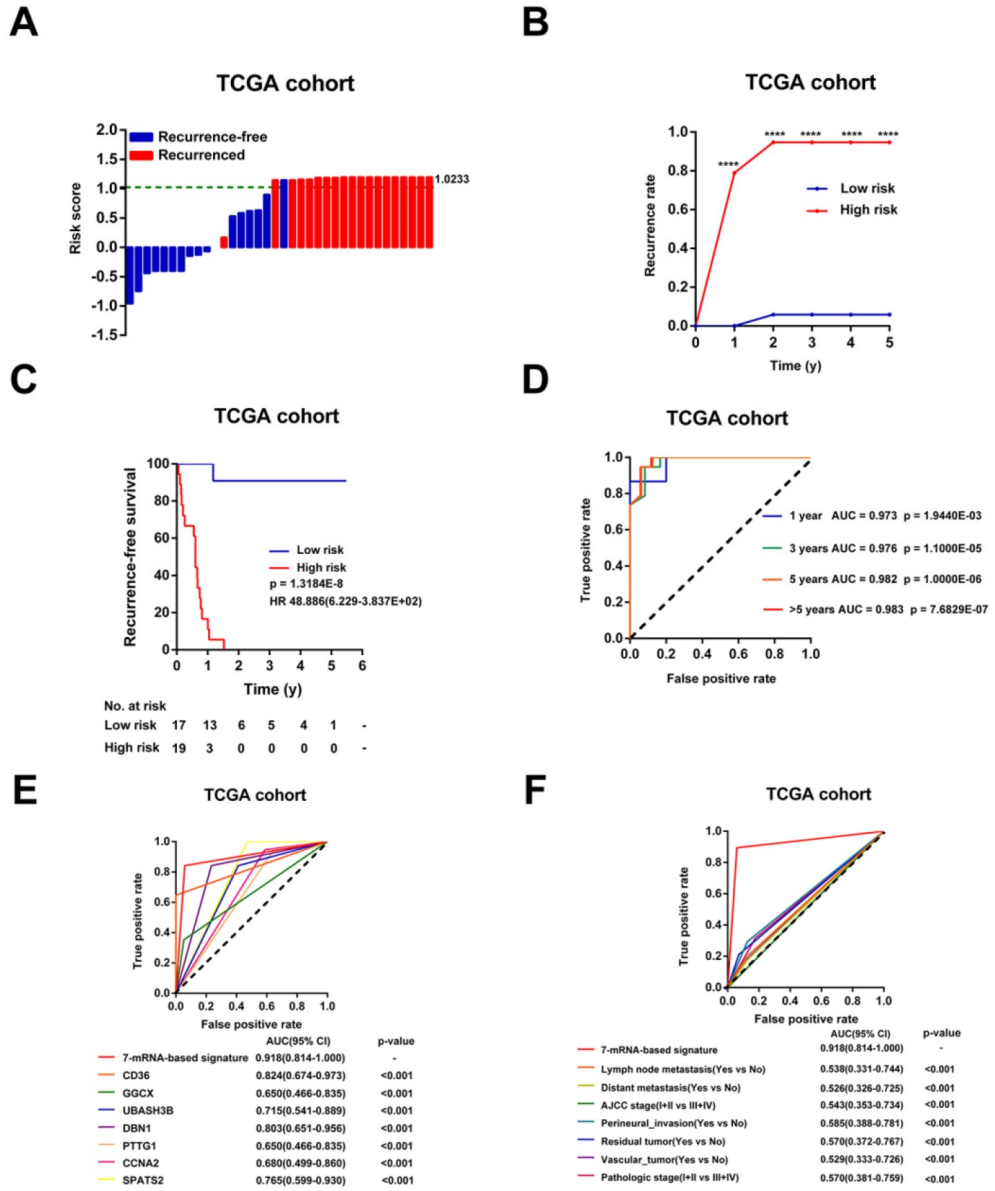
Evaluation of the risk score formula for relapse in the TCGA cohort. (A) Waterfall plots for distribution of risk score and relapse status of individual patients. (B) Recurrence rate between the high- and low-risk at the indicated time. (C) The Kaplan-Meier survival curve of recurrence-free for patients between two different groups. (D) Time-dependent ROC curve at 1 year, 3 years, 5 years and more than 5 years. (E) Comparison of prognostic accuracy between the signature and single mRNAs. (F) Comparison of prognostic accuracy between the signature and clinical characteristics. P-values were calculated using the log-rank test. HR, hazard ratio; AUC, area under ROC curve; RFS, recurrence-free survival. ****, p <0.001.

Then, the risk scores for relapse were calculated for every patient in the TCGA Cohort. As shown in Figure 4A, the patients were more trended to relapse when the risk score increased. Patients were divided into high-risk (n=17) or low-risk (n=19) groups using the optimal risk score as the cutoff point. The Recurrence rate of the high-risk group was extremely increased throughout the study period until the analytical endpoint, at which 94.74% of patients in the high-risk group experienced CCA recurrence while only 0.58% of patients in low-risk group relapsed (p < 0.001, Figure 4B). Kaplan-Meier analysis showed that CCA patients with higher risk score had significantly worse RFS than those with lower risk score (HR = 48.886, 95% CI: 6.229-383.657, p < 0.001, Figure 4C). In addition, the time-dependent ROC curves between the 7-mRNA signature and RFS showed that AUC at 1 year, 3 years, 5 years, and > 5 years were 0.973, 0.976, 0.982 and 0.983, respectively (all p < 0.001, Figure 4D). Besides, compared with any single mRNA or clinical factors, the 7-mRNA-signature had better predictive value for relapse (all p < 0.001, Figure 4E-F).

In addition, univariable Cox analysis showed that only 7-mRNA signature were positively associated with CCA recurrence in TCGA cohort (p < 0.001, HR = 48.886, 95% CI = 6.229-383.657, Table S3). However, clinical association analyses showed that increased risk score was not related to clinical factors obviously, probably due to the small sample numbers (Table S4).

### Validation of the 7-mRNA signature for recurrence-free survival prediction in Ren Ji Cohort

To further verify whether this 7-mRNA classifier had a similar predictive ability in different CCA populations, we applied it to an independent cohort. From January 2012 to December 2017, forty-four CCA patients with complete clinicopathological information and prognostic outcomes were enrolled in our study at Ren Ji Hospital. We measured the expression levels of the 7 mRNAs in 44 CCA tumor samples by qRT-PCR assays (Figure S4). Then, the risk scores were calculated for every patient according to the expression status of these 7 mRNAs.

**Figure 5:**
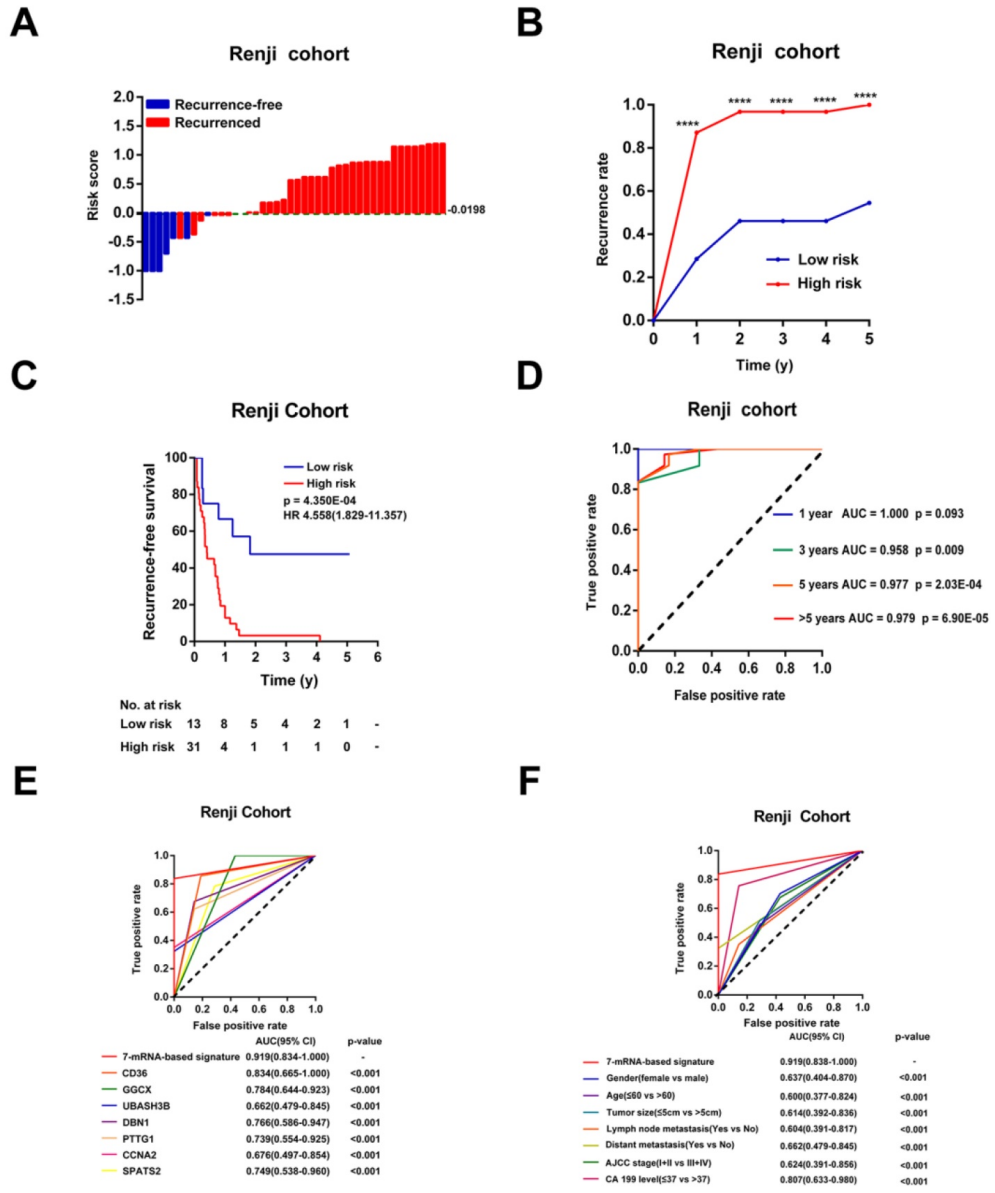
Validation of the 7-mRNA signature for relapse prediction in the Ren Ji cohort. (A) Waterfall plots for distribution of risk score and relapse status of individual patients. (B) Recurrence rate between the high- and low-risk at the indicated time. (C) The Kaplan-Meier survival curve of recurrence-free for patients between two different groups. (D) Time-dependent ROC curve at 1 year, 3 years, 5 years and more than 5 years. (E) Comparison of prognostic accuracy between the signature and single mRNAs. (F) Comparison of prognostic accuracy between the signature and clinical characteristics. P-values were calculated using the log-rank test. HR, hazard ratio; AUC, area under ROC curve; RFS, recurrence-free survival. ****, p <0.001.

According to the optimal cutoff risk score determined by the ROC curve, patients were further divided into high- (n = 31) or low-risk (n = 13) groups. As shown in Figure 5A-B, patients with higher risk scores were more prone to recurrence after CCA resection. Survival analysis showed that patients in the high-risk group had obviously shorter RFS time than those in the low-risk group (p < 0.001, HR = 4.558, 95% CI 1.829-11.357, Figure 5C). The AUCs of the time-dependent ROC curves between the 7-mRNA signature and RFS were 1.000 for 1 year, 0.958 for 3 years, 0.977 for 5 years and 0.979 for >5 years (p = 0.09 at 1 year, others p < 0.01, Figure 5D). Moreover, the AUC of the 7-mRNA risk score model was significantly greater than any single mRNA or clinical factor (all p<0.001, Figure 5E-F).

Univariable Cox analyses of the Ren Ji cohort showed that CA19-9 levels, lymph node metastasis, and the 7-mRNA signature were significant factors that correlated with RFS of CCA (Table S5). Among these, the 7-mRNA signature was the most effective one to predict relapse of CCA in the Ren Ji cohort (p = 0.001, HR = 4.558, 95% CI 1.829-11.357, Table S5). Furthermore, the multivariable Cox analysis showed that the 7-mRNA signature remained a powerful and independent factor for RFS after adjusting for other clinicopathological characteristics (p = 0.008, HR = 3.912, 95% CI = 1.417-10.799, Table S5). In addition, the 7-mRNA signature was found to be positively associated with the tumor size (p = 0.034) of CCA (Table S6).

### Stratification analysis of the 7-mRNA-based classifier in TCGA Cohort and Ren Ji Cohort

**Figure 6:**
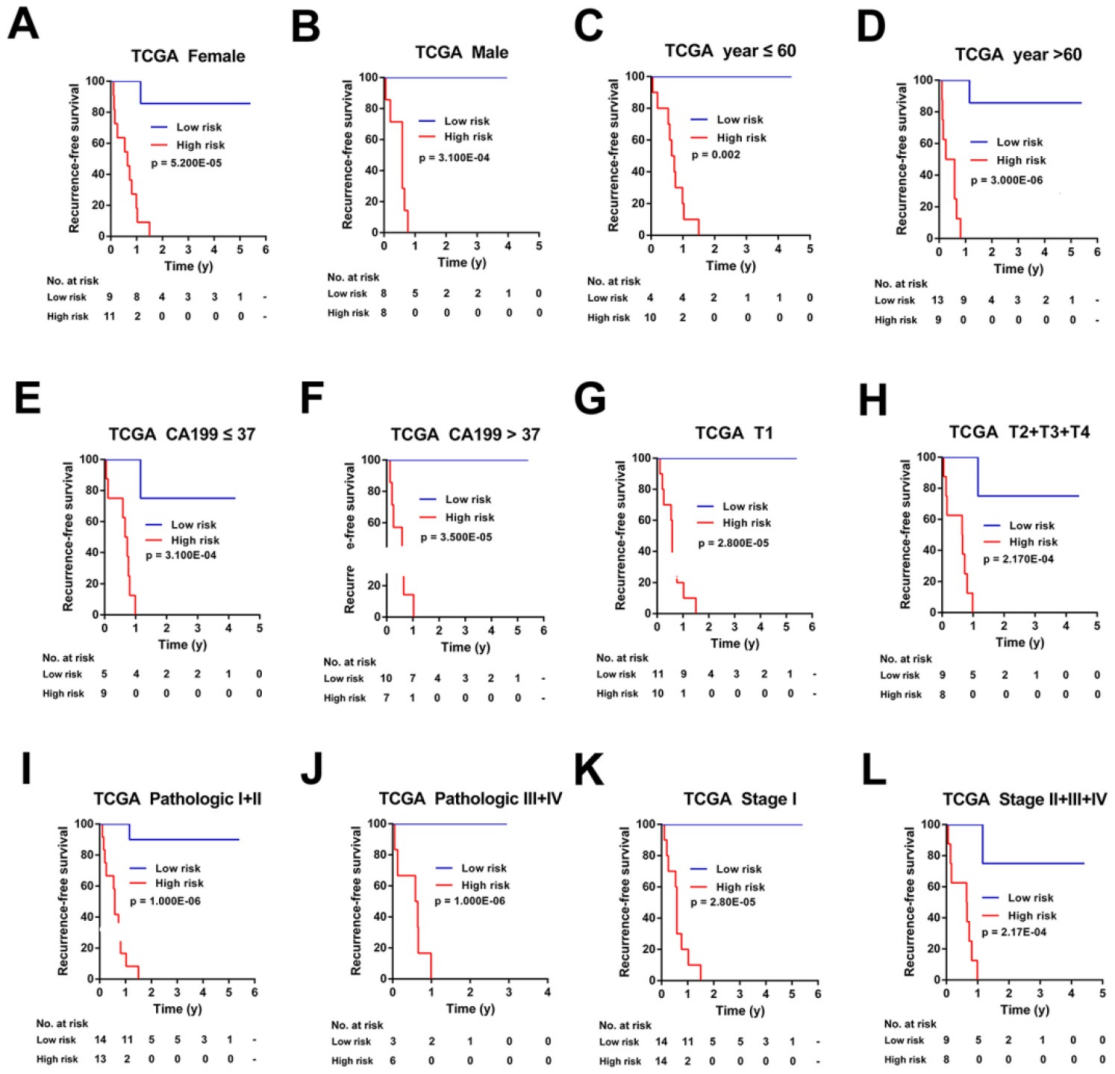
Kaplan-Meier survival analyses of the TCGA cohort, according to the 7-mRNA-based classifier stratified by clinicopathological characteristics. (A, B) Gender, (C, D) Age, (E, F) CA 199 levels, (G, H) Tumor size, (I, J) Pathologic stage, and (K, L) AJCC stage.

**Figure 7:**
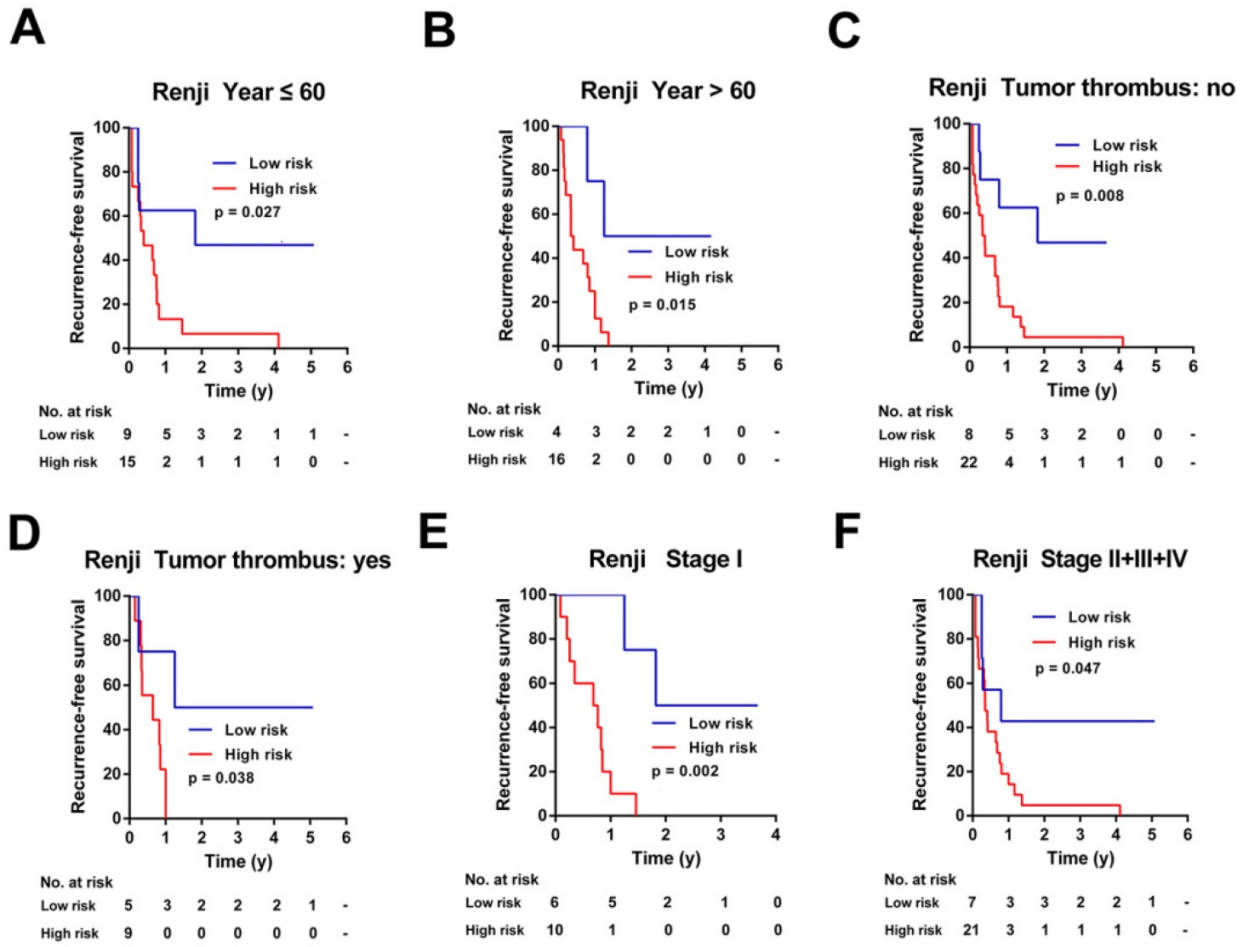
Kaplan-Meier survival analyses of the Ren Ji cohort, according to the 7-mRNA-based classifier stratified by clinicopathological characteristics. (A, B) Age, (C, D) Tumor thrombus, and (E, F) AJCC stage.

To investigate the applicable CCA population of this 7-mRNA-based classifier, the 7-mRNA-signature based survival analyses were further performed in subgroups of patients with different clinical variables in the TCGA cohort and Ren Ji cohort (Figure 6-7, Figure S5-6).

For the TCGA cohort, upon stratified by individual clinicopathological features including gender, age, CA19-9 levels, tumor size, pathologic stage, and AJCC stage, the signature was still a clinically and statistically significant applicable model in predicting recurrence of CCA patients (Figure 6). However, because of the small sample size, for patients with positive of lymph node metastasis, distant metastasis, perineural invasion, residual tumor or vascular tumor, the 7-mRNA signature was a little powerless for relapse prediction (Figure S5).

Similarly, this 7-mRNA signature was a practical predictor that was independent of some clinicopathological characteristics like age, tumor thrombus and AJCC stage in the Renji cohort (Figure 7). For patients in subgroups of male, CA19-9 ≤ 37ng/ml, tumor size ≤ 5cm, mono-modular, negative of lymph node metastasis or distant metastasis, the 7-mRNA signature maintained its predictive value for recurrence-free survival (Figure S6B, S6C, S6E, S6G, S6I, and S6K). Unfortunately, the 7-mRNA signature lost the prognostic role for patients of female, CA19-9 > 37ng/ml, tumor size > 5cm, multi-modular, positive of lymph node metastasis or positive of distant metastasis, which might be due to the small sample number of these subgroups (Figure S6A, S6D, S6F, S6H, S6J and S6L).

## Discussion

Cholangiocarcinoma is a fatal malignancy, which arising from varying locations within the biliary tree. Although surgical resection with curative intent is performed, the prognosis of patients with CCA remains poor owing to a high incidence of recurrence. Therefore, predicting recurrence is an arduous and urgent task 20.

At present, tumor biomarkers have been used to predict relapse in patients with CCA. Firstly, Carbohydrate antigen 19-9 (CA19-9) is a traditional serum biomarker used for CCA prognosis prediction. Some studies found that preoperative CA19-9 level higher than 100U/ml were associated with a lower recurrence-free survival after operation. However, these tumor markers are not specific to CCA, and its elevation can be related to other diseases, such as bile duct obstruction or acute cholangitis 21. Another study pointed out that preoperative serum CA19-9 level higher than 135U/ml was a predictor for a lower survival rate 22. Other serum markers, such as carcinoembryonic antigen (CEA), have proved to be overlapped with other diseases and showed low sensitivity and specificity. It has been reported in many studies that serum Cytokeratin 19 fragment 21-1 (CYFRA21-1) and CA-242 have higher specificities than CA19-9 for intrahepatic cholangiocarcinoma, but they haven't been used in clinical routine examination 23. Some clinical features, such as tumor size and lymph node metastases, remain controversial in relapse prediction 24-26.

Currently, different versions of the AJCC or TNM staging system, along with the prognostic scoring systems have been widely used to evaluate the prognosis of CCA patients. However, these systems had some limitations in different subtypes of CCA 5, which might due to the ignorance of the different genetic and epigenetic backgrounds in tumor subtypes. In summary, postoperative prediction of CCA remains a problem, so, we conducted a multi-mRNA signature to accurately predict RFS for CCA patients.

In this study, we firstly selected datasets from GEO and TCGA. Then, we performed overlapping analysis with a strategic and stepwise method and finally obtain 194 DEGs in accordance with uniform standards (p < 0.01 and FC >1.5 or <-1.5). After screening, 127 DEGs with AUC ≥ 0.55 were utilized to construct the LASSO COX regression model. Ten-fold cross-validation was used to select the best one with the minimum mean cross validation error from a series of models. Finally, we established a 7-mRNA-based signature risk score model for CCA patients. Cox univariable and multivariate analysis verified that 7-mRNA-signature was a powerful and independent prognostic factor for CCA patients. The time-dependent ROC curve demonstrated that this model was superior to other prognostic factors, such as CA19-9 levels and the AJCC staging system.

Meanwhile, we used two datasets to validate prognostic value of 7-mRNA-signature in relapse. The results proved that patients in the high-risk group tended to recurrence compared with the low-risk group.

Most genes included in the 7-mRNA signature have been experimentally researched in many studies. Uray et al. found that higher expression of CD36 could mediate TSP-1-stimulated apoptosis and promoted cellular adhesion in breast cancer cells, which might inhibit tumor migration and invasion in cholangiocarcinoma 27. However, CD36 was a controversial indicator in pancreatic cancer, low expression of CD36 predicted lower TNM staging and CA19-9 levels, but larger tumor size and poor survival prognosis 28. Ueda *et al.* reported that exon 2 deletion splice variant of GGCX could result in des-γ-carboxy prothrombin (DCP) production in HCC cell lines 29, so, we guessed that GGCX metabolite DCP could play the same role in cholangiocarcinoma. Lee et al. found that phosphatase activity of UBASH3B could improve EGFR protein abundance, invasion, and metastasis in TNBC 30, and EGFR also played an important role in the progression of cholangiocarcinoma 1. Iyama *et al.* reported that overexpression of DBN1 was associated with poor outcome of lung adenocarcinoma 31. Meanwhile, DBN1 was reported to be involved in actin cytoskeleton reorganization, which had a vital role during cancer metastasis 32. Ren *et al.* verified that pituitary tumor transforming gene-1 (PTTG1) was an independent prognostic factor and acted as an oncogene in colorectal cancer 33. Furthermore, PTTG1 was known as a transcription factor, which exerts transcriptional activity either by directly binding to DNA or by interacting with proteins, including p53 34, and p53 was consistently an important cancer suppressor gene in cholangiocarcinoma 35. Li *et al.* found that cyclin A2 (CCNA2) promoted the EMT progression combined with MET/AKT/GSK-3b via the ROCK/AKT/β-catenin pathway in bladder cancer 36, and aberrant activation of Wnt/β-catenin signaling was observed in the majority of CCA 37. In colorectal cancer, knockdown of CCNA2 inhibited cancer growth by impairing cell cycle 38***.*** Up-regulation of SPATS2 expression activated the STAT3 pathway and resulted in poor prognosis 39, and STAT3 had been implicated in carcinogenesis 40. Besides, Takamochi *et al.* found that SPATS2 could help to differentiate squamous cell carcinoma from adenocarcinoma of the lung 41. Unfortunately, the association of these 7 genes and cholangiocarcinoma recurrence has not been reported until now. However, CD36, GGCX, UBASH3B, DBN1, PTTG1, CCNA2, and SPATS2 are found in other tumors to regulate tumor progression, which may also regulate cholangiocarcinoma progression and affect the recurrence of CCA. Therefore, the potential mechanisms of these 7 genes with CCA recurrence need to be studied furthermore.

However, there were still some limitations to our study. Firstly, we used a relatively small sample size of CCAs. Secondly, besides mRNA, the predictive value of microRNA, lncRNA, and CpG in tumor prognosis had been validated. Multi-dimensional data analysis integrated with mRNA, microRNA, lncRNA, CpG might further increase the predictive efficiency 42-44. Finally, cell functions and molecule mechanisms for the 7 mRNAs had not been explored in our study.

## Supplementary Material

Supplementary figures and tables.

## Figures and Tables

**Table 1:** GEO datasets enrolled in the study.

Database	Source	Sample			Platform
		T	P	N	
**GSE26566**	https://www.ncbi.nlm.nih.gov/geo/query/acc.cgi?acc=GSE26566	106	59	6	Illumina v2.0
**GSE32879**	https://www.ncbi.nlm.nih.gov/geo/query/acc.cgi?acc=GSE32879	16	-	7	Affymetrix 1.0 ST
**GSE76297**	https://www.ncbi.nlm.nih.gov/geo/query/acc.cgi?acc=GSE76297	91	92	-	Affymetrix HTA-2_0
**GSE45001**	https://www.ncbi.nlm.nih.gov/geo/query/acc.cgi?acc=GSE45001	10	10	-	Agilent-028004
**GSE31370**	https://www.ncbi.nlm.nih.gov/geo/query/acc.cgi?acc=GSE31370	6	-	5	Illumina V4.0

T: tumor tissues; P: para-cancerous tissues; N: normal intrahepatic bile duct tissues or non-tumor tissues.

## Abbreviations

mRNA: messenger RNA
CCA: Cholangiocarcinoma
GEO: Gene Expression Omnibus
TCGA: The Cancer Genome Atlas
LASSO: Least absolute shrinkage and selection operator
HR: hazard ratio
CI: confidence interval
AJCC: American Joint Committee on Cancer
KRAS: KRAS proto-oncogene GTPas
EGFR: epidermal growth factor receptor
ERBB2: erb-b2 receptor tyrosine kinase 2
ROC: receiver operating characteristic
RFS: recurrence-free survival
GO: gene ontology
KEGG: Kyoto Encyclopedia of Genes and Genomes
DEGs: differentially expressed genes
FC: fold change
BP: biological processes
CC: cellular component
MF: molecular function
AUC: Area Under Curve
PCR: Polymerase Chain Reaction
CD36: CD36 molecule
GGCX: Gamma-glutamyl carboxylase
UBASH3B: ubiquitin associated and SH3 domain containing B
DBN1: drebrin 1
PTTG1: pituitary tumor transforming gene-1
CCNA2: cyclin A2
SPATS2: spermatogenesis associated serine rich 2
CA19-9: Carbohydrate antigen 19-9
PSC: primary sclerosing cholangitis
CEA: carcinoembryonic antigen
CYFRA21-1: Cytokeratin 19 fragment 21-1
