# Supplementary material for: Prognostic values of a novel multi-mRNA signature for predicting relapse of cholangiocarcinoma
Source: Int J Biol Sci. 2020 Jan 16;16(5):869–81. doi: 10.7150/ijbs.38846 (PMC7019144; doi:10.7150/ijbs.38846)
Supplement: Supplementary file 1 — Supplementary figures and tables. [file ijbsv16p0869s1.pdf]

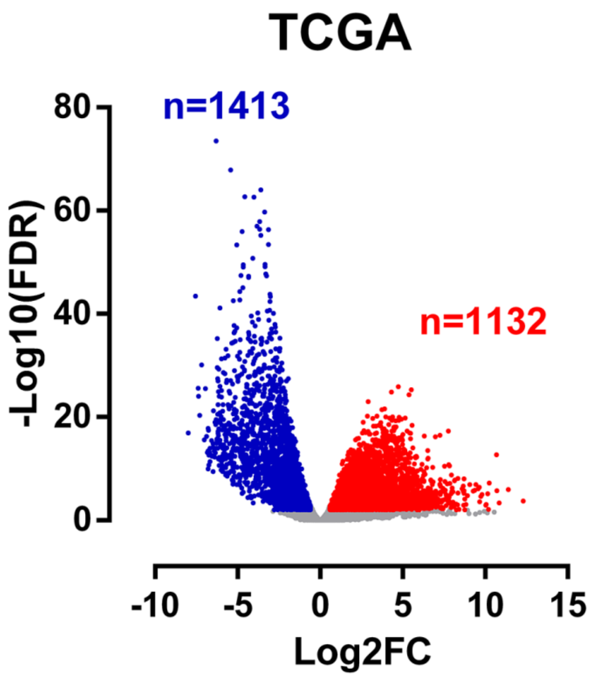

Fig.S1 Volcano plots of DEGs in TCGA cohort. (X-axis: log[2](FC); Y-axis: -log[10](FDR) for each gene. Genes with FDR < 0.01 and FC>1.5 or <-1.5 were considered as DEGs. Blue: down-regulated genes; Grey: non-differential genes; Red: up-regulated genes.

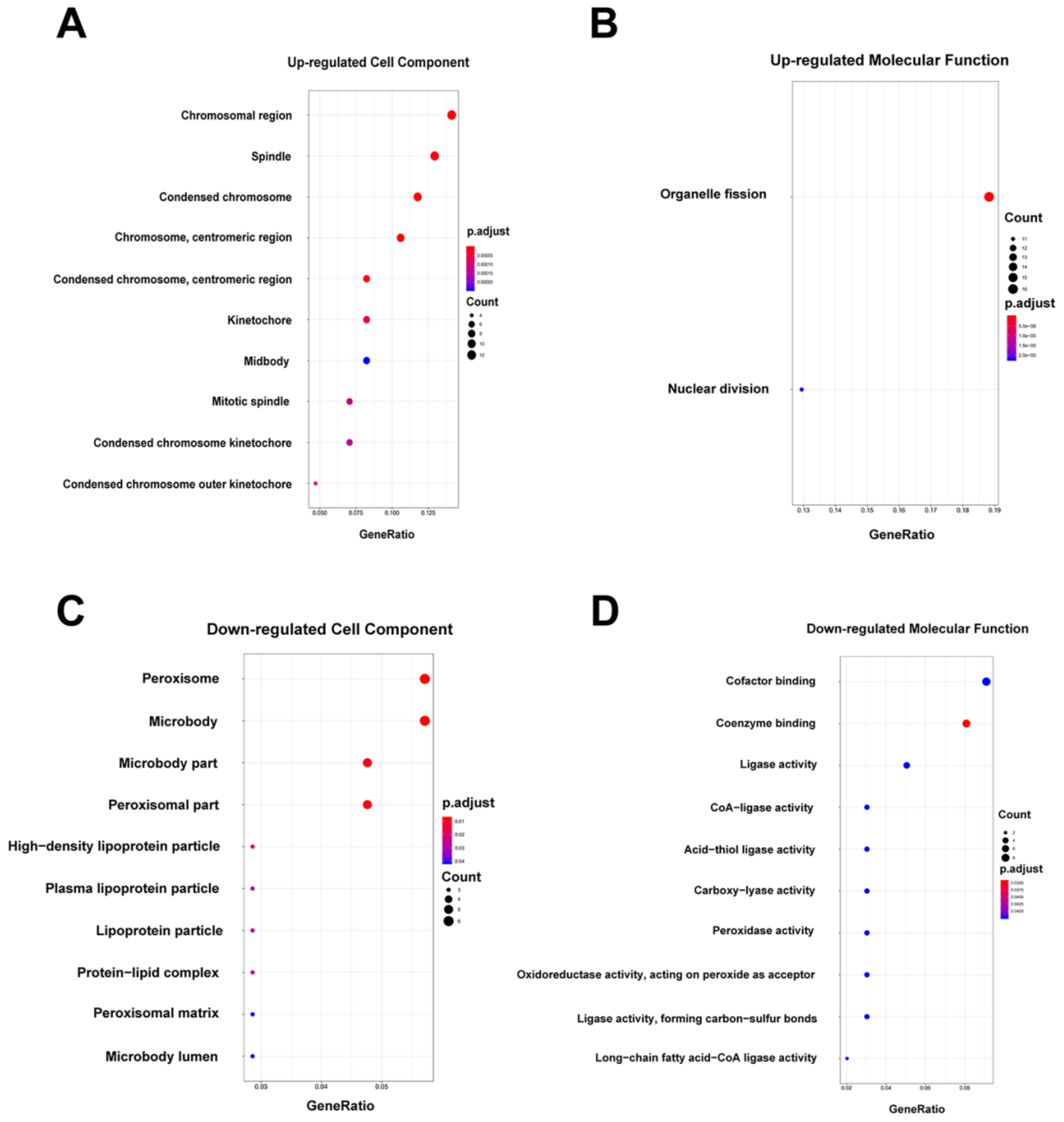

Fig.S2 GO cell component (CC) enrichment analysis of upregulated DEGs (A) and downregulated DEGs (C). GO molecular function (MF) analysis of upregulated DEGs (B) and downregulated DEGs (D).

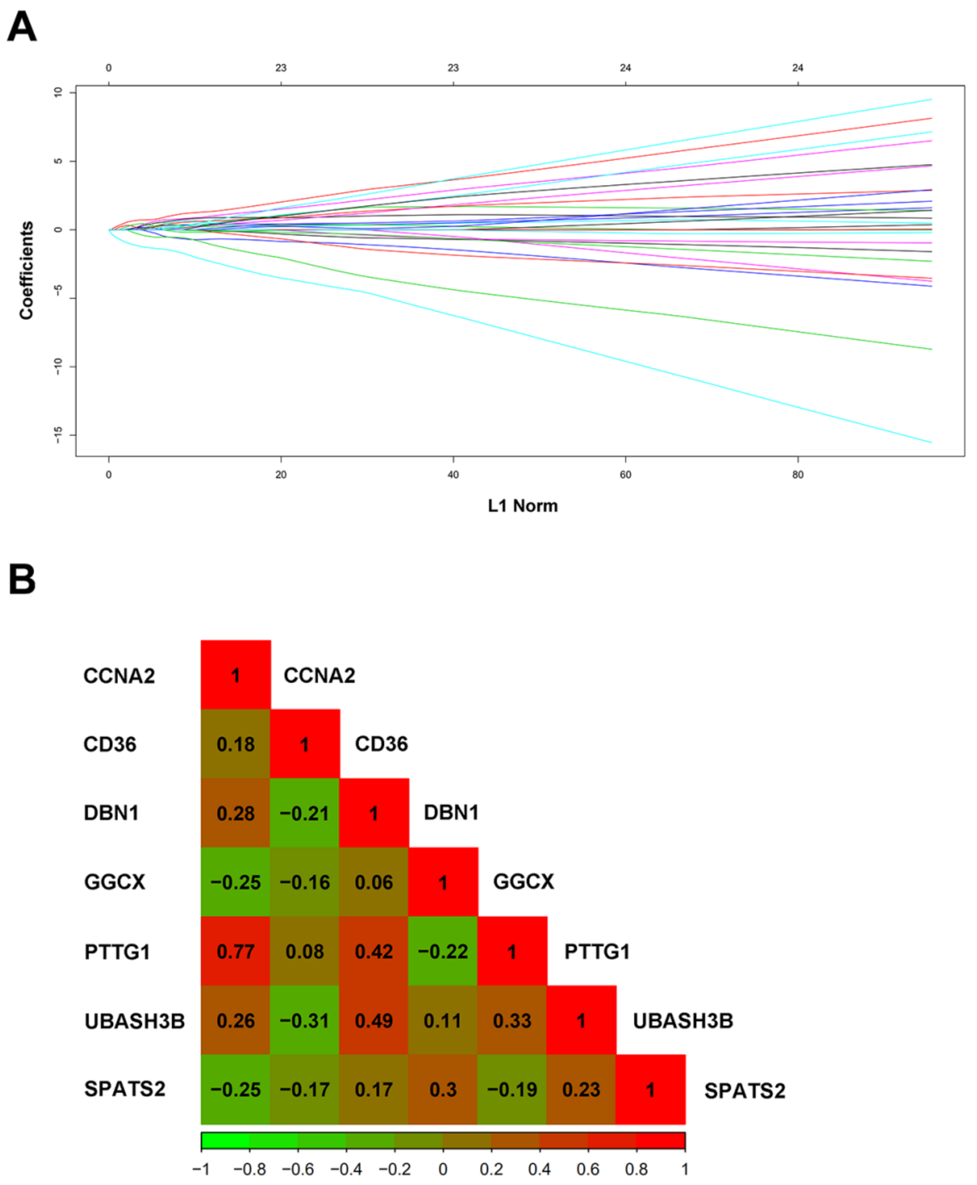

Fig.S3 Complete LASSO coefficient profiles of the 127 mRNAs. Each curve represents a variable. The above axis: the number of nonzero coefficients at as λ varies. X-axis: L1 Norm, the summation of absolute nonzero coefficients at as λ varies. Y-axis: the values of nonzero coefficients at as λ varies.

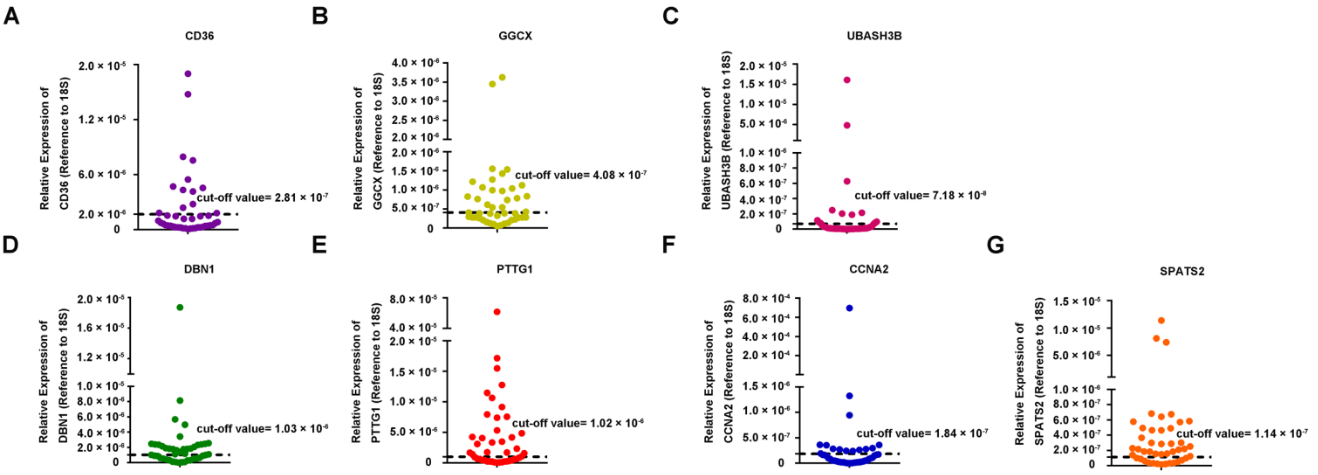

Fig.S4 mRNA expression levels of 7 genes in 44 CCA samples from Renji hospital and each cut-off value were shown in figure. (A-G)

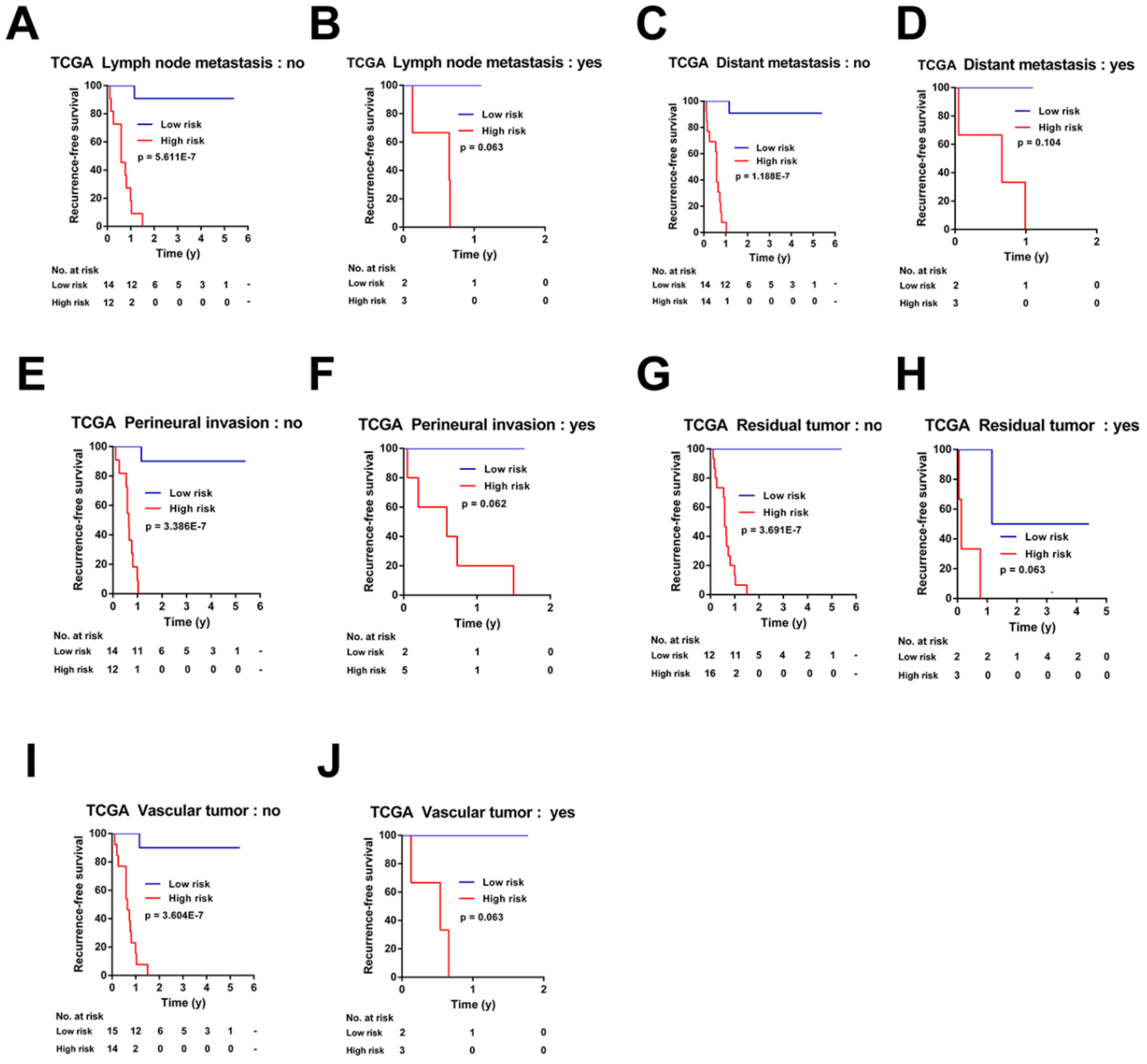

Fig.S5 Kaplan-Meier survival analyses of the TCGA cohort, according to the 7-mRNA-based classifier stratified by clinicopathological risk factors. (A, B) Lymph node metastasis, (C, D) Distant metastasis, (E, F) perineural invasion, (G, H) Residual tumor, and (I, J) Vascular tumor.

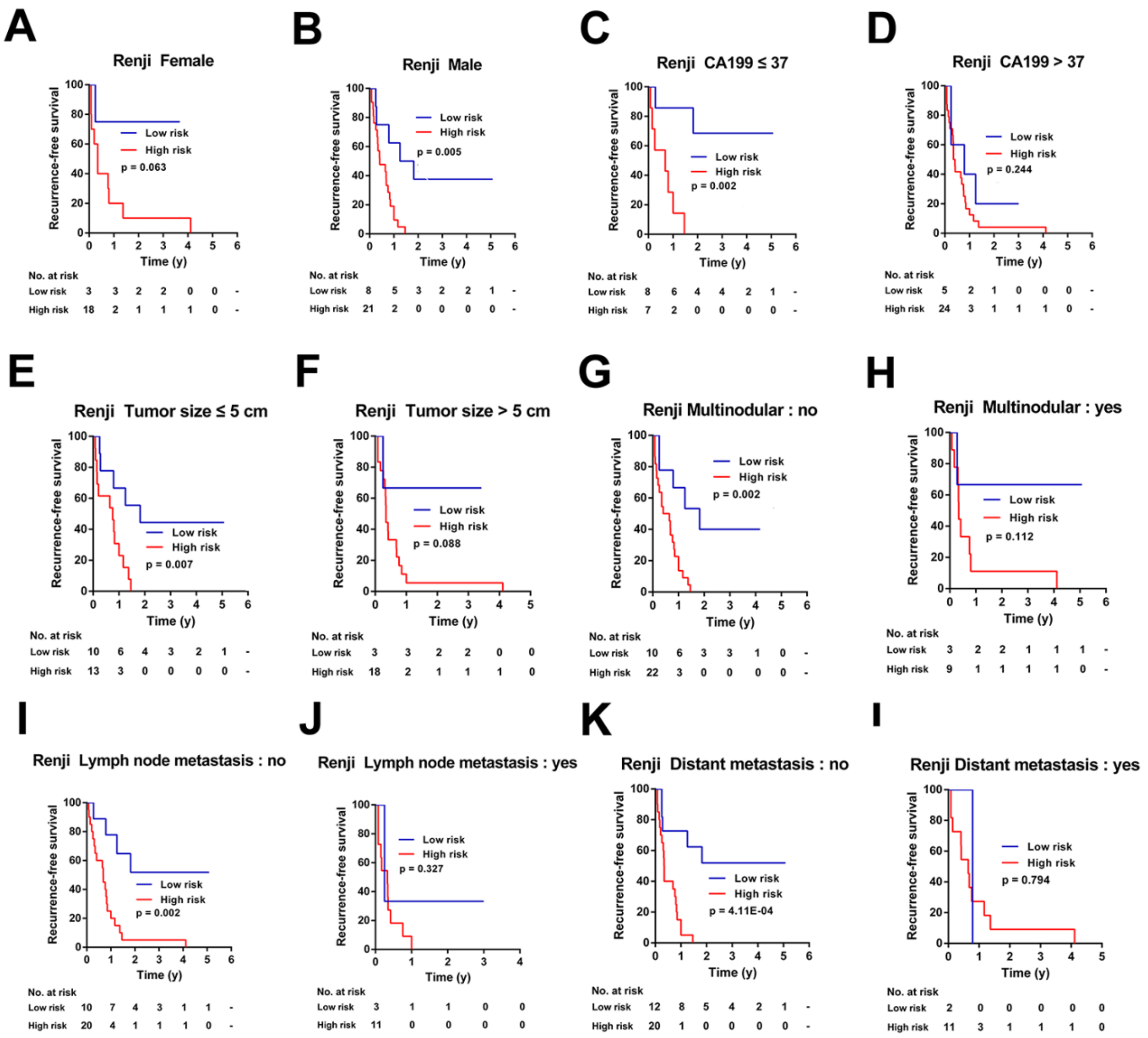

Fig-S6 Kaplan-Meier survival analyses of the Ren Ji cohort, according to the 7-mRNA-based classifier stratified by clinicopathological risk factors. (A, B) Gender, (C, D) Age, (E, F) Tumor size, (G, H) Multinodular, (I, J) Lymph node metastasis, and (K, L) Distant metastasis.
